# Epibiont Cohabitation in Freshwater Shrimp *Neocaridina davidi* with the Description of Two Species New to Science, *Cladogonium kumaki* sp. nov. and *Monodiscus kumaki* sp. nov., and Redescription of *Scutariella japonica* and *Holtodrilus truncatus* †

**DOI:** 10.3390/ani13101616

**Published:** 2023-05-12

**Authors:** Rafał Maciaszek, Wiesław Świderek, Sebastian Prati, Chih-Yang Huang, Kamil Karaban, Anita Kaliszewicz, Aleksandra Jabłońska

**Affiliations:** 1 Department of Animal Genetics and Conservation, Institute of Animal Sciences, Warsaw University of Life Sciences, ul. Ciszewskiego 8, 02-786 Warsaw, Poland; 2 Department of Aquatic Ecology, University of Duisburg-Essen, Universitätstr. 5, 45141 Essen, Germany; 3 Department of Aquaculture, National Taiwan Ocean University, 2 Beining Road, Jhongjheng, Keelung 202301, Taiwan; 4 Institute of Biological Sciences, Cardinal Stefan Wyszynski University in Warsaw, ul. Wóycickiego 1/3, 01-938 Warsaw, Poland; 5 Department of Invertebrate Zoology and Hydrobiology, University of Lodz, ul. Banacha 12/16, 90-237 Łodź, Poland

**Keywords:** ornamental pet trade, aquarium crustacean, Pithophoraceae, Temnocephalidae, Branchiobdellida

## Abstract

**Simple Summary:**

Examination of epibiont distribution in *Neocaridina davidi* shrimp revealed occurrence of two species new to science. The number of epibionts differed between the four designated microhabitats on the host body, as well as between wild, aquaculture, and aquarium shrimp, most likely reflecting their preferences. Some epibiotic species may adversely affect the host, while others can be used to control others. Thus, the ability to identify them, as well as knowledge of their preferences and impact on the host and each other, may prove useful in controlling their spread and limiting accidental co-introductions as potentially invasive alien species.

**Abstract:**

This contribution presents the occurrence of epibiotic species associated with *Neocaridina davidi* shrimp collected in the wild, aquaculture ponds, and aquaria. A total of 900 shrimp are imported from Taiwan, three-quarters of which host at least one of the recorded epibionts. Among those epibionts, two species new to science are discovered, *Cladogonium kumaki* sp. nov. and *Monodiscus kumaki* sp. nov., while the other two, *Holtodrilus truncatus* and *Scutariella japonica*, are redescribed. The largest number of epibionts is found in shrimp collected from aquaculture ponds and the lowest in individuals from aquaria. Epibiont occurrence differs across designated microhabitats. The epibionts may be introduced alongside their host outside their native range, and their presence may affect shrimp breeding rates. Thus, more control over them should be provided. Their spread can be limited by removal from the host during molting or manually, as well as by using interspecies interactions.

## 1. Introduction

Releases of aquatic ornamental species, such as crustaceans, are considered one of the main introductory pathways of alien species worldwide [1,2,3,4]. Once introduced into habitats outside their native range, regardless of their ability to establish, they may directly or indirectly have a negative impact on natural ecosystems, which can lead to biodiversity loss [5]. The impact on native biota may be further exacerbated by the cointroduction of associated organisms, such as epibionts [3,6].

Among freshwater ornamental shrimps sold in the global aquarium trade, *Neocaridina davidi* (Bouvier, 1904) is one of the most common. The shrimp gained great popularity because of its many color varieties and relatively easy breeding [7,8]. This popularity led to its mass production in aquaculture ponds [9]. Nowadays, the species is exported worldwide, mainly for the private aquaria of shrimp enthusiasts. However, not all the *N. davidi* available in the trade originate from aquaculture farms, as wild-caught shrimp are also sought after. Wild, less-colored shrimp are mainly used as cheap algae-eaters in aquaria and a live source of food for various aquarium species, including fish and other crustaceans (crayfish, crabs, and other shrimps) [10]. This shrimp is not endangered or protected; hence, wild individuals are common in the pet trade [11]. Wild shrimp are collected in a wide variety of habitats across their native range. Thus, they might potentially host a wider array of associated biota compared to farmed ones, increasing chances of cointroduction into novel environments once released.

Due to intentional releases, *N. davidi* has already established itself in several locations beyond its native range [7,12,13,14,15]. In a few instances, non-native *N. davidi* hosted alien epibionts such as the branchiobdellidan *Holtodrilus truncatus* (Liang, 1963) and the temnocephalid *Scutariella japonica* (Matjašič, 1990) [16,17,18]. More recently, microsporidians, which are intracellular parasites, have been found in a European population of *N. davidi* [19]. However, *N. davidi* is known to host other epibiotic species, which has not yet been reported from feral populations. These include bacteria, ciliates, rotifers, and pithophoraceans [9,20,21]. Nevertheless, as noted by Patoka et al. [3], the introduction pathway of organisms associated with freshwater shrimps via the international pet trade has been, so far, poorly evaluated.

In this study, we screened randomly collected *N. davidi* individuals for the presence of epibionts belonging to the following groups: Chlorophyta: Pithophoraceae, Platyhelminthes: Temnocephalidae, and Annelida: Branchiobdellidae. The aim was to identify epibiont species, their prevalence, and possible co-occurrence on *N. davidi* shrimp originating from the wild, aquaculture ponds, and aquarium farms. Moreover, epibionts’ microhabitat preferences and their potential reciprocal interactions were also investigated.

## 2. Materials and Methods

### 2.1. Preliminary Study

In response to the concerns of Taiwanese aquarium shrimp breeders relative to the occurrence of epibionts, in October 2018, we screened shrimp from three different potential sources: wild, aquaculture ponds, and aquaria. First, we investigated 100 *N. davidi* shrimp randomly collected using aquarium fish nets from the Wai Shuang Hsi River in Taipei City, Taiwan (25.0624° N, 121.3327° E), randomly collected by local breeders using aquarium fish nets. These shrimp are commonly used as a live source of food for aquarium pets. Afterward, we investigated shrimp bred for aquarium purposes in aquaculture ponds and aquaria (100 individuals each) in Pingtung County, Taiwan. They were randomly collected with nets by their owners. In each case, the shrimp were examined in situ for the presence of epibionts. If detected, they were observed and photographed under a stereoscopic microscope.

### 2.2. Animals Collection from the Aquarium Trade

For further investigation, a total of 900 *N. davidi* shrimp were imported from Taiwan. Of these, 300 were wild caught from three localities (Table 1), 300 were derived from aquaculture ponds, and 300 from aquaria. For each of the three environments, 100 shrimp were collected in three independent locations to ensure replicates. Individuals were collected using aquarium fish nets by local shrimp breeders in November 2018 (within a few days of exporting to Poland), and the size was selected in situ (2.0 ± 0.17 cm) with the sieve normally used in shrimp aquaculture farms. Shrimp were packed in 12 L aquarium bags filled with 50% fresh habitat water and 50% oxygen. They were put inside Styropor boxes and exported via air cargo to Poland for further examination, passing through all veterinary inspections required for aquarium pets (Taipei, TW; Amsterdam, NL/EU; Warsaw, PL/EU). The Styropor boxes were then collected from Warsaw Chopin Airport and transported to the private laboratory of an ornamental shrimp farm (Kumak Shrimp, Konstancin-Jeziorna, Poland). The water temperature was maintained between 20 and 25 °C during transport from the sampling sites in Taiwan to Poland.

### 2.3. Epibiont Detection and Identification

**Figure 1:**
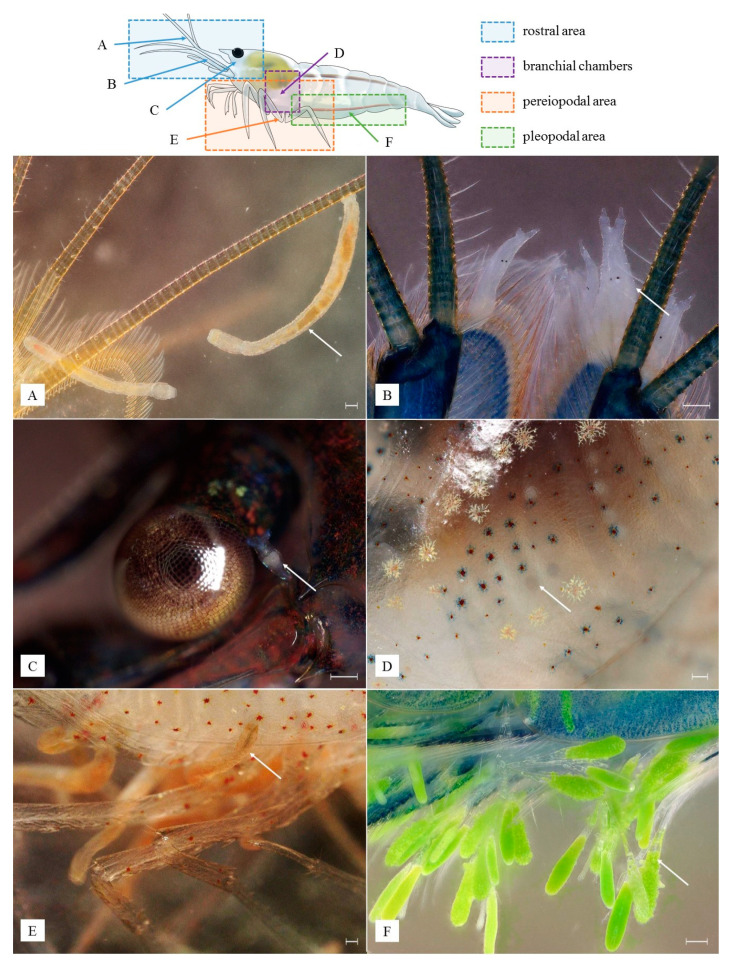
Epibionts observed in *Neocaridina davidi*: rostral area (**A**–**C**) inhabited by *Holtodrilus truncatus* (**A**), *Scutariella japonica* (**B**)*,* and bdelloid rotifer (**C**); branchial chambers with deposited eggs of *Monodiscus kumaki* sp. nov. (**D**); pereiopodal area colonized by *H. truncatus* (**E**); pleopodal area with *Cladogonium kumaki* sp. nov. (**F**); white scale bar = 100 µm. (Photographs and drawings by R. Maciaszek).

After arrival, the shrimp were acclimatized in aquaria of 20 × 20 × 25 cm, one for each transport bag, filled with a similar amount of water from the transport bags (∼6 L) and illuminated using 6500 K 21 W fluorescent lamps. After 10 h, the shrimp were individually analyzed for the presence of epibionts or traces of their activity in four different microhabitats, defined according to Maciaszek et al. [9] as (1) rostral area, (2) branchial chambers, (3) pereiopodal area, and (4) pleopodal area (Figure 1). The presence of ciliates and rotifers was used as an indicator of organic matter in the water and food source for temnocephalids and branchiobdellidans [9,22,23]. Preliminary observations were carried out directly in each aquarium by secluding each shrimp with a petri dish pushed against the inner part of the tank wall, as shown in Maciaszek et al. [24]. The shrimp were separated and put individually in 50 mL plastic containers filled with aquarium water to curb the movement of epibionts between shrimp and facilitate further inspection under the microscope.

Epibionts were isolated from live shrimp sedated using clove oil (nine parts of ethyl alcohol and one part of eugenol) well-dissolved in aquaria water. If restless, branchiobdellidans and temnocephalids were additionally sedated. Clove oil was gradually added drop by drop at 60 s intervals, depending on the shrimp or the size of the epibiont and their reaction. Epibionts were then dorsally, ventrally, and laterally observed and morphologically identified. In the case of pithophoraceans, single individuals were removed carefully from the body of shrimps. Nuclei in their erect cells were observed using iodine (one part of iodine to three parts of water). Shrimp were then put in 60 L quarantine tanks filled with aquarium water for two-week observations of treatment impact on their survival and condition.

Morphological identification was carried out using available literature [21,22,25,26,27] under Leica DM50000B (Leica Camera AG, Wetzlar, Germany) and Keyence VHX-7000 (Keyence Corporation, Osaka, Japan) microscopes at 500–1500× magnification at the Institute of Biological Sciences, Cardinal Stefan Wyszynski University in Warsaw. Epibionts were measured under the microscope using dedicated software. All photographs were taken with a Canon EOS 5D connected to the microscope via a DR-E 6 DC coupler (Canon Inc., Tokio, Japan).

### 2.4. Data Analysis

All statistical and descriptive analyses were performed with open-source software RStudio version 2022.12.0 (RStudio Inc., Boston, MA, USA) based on R version 4.2.2 [28]. Before proceeding with analyses relative to epibionts, the sex ratios in *N. davidi* were compared among the three sources (wild, aquaculture ponds, and aquaria) using the Chi-square test, followed by a Bonferroni-adjusted pairwise test of independence. This was done to ensure that the sex ratio differences among the sources did not bias the results.

The prevalence and mean intensity of epibionts were then compared among females and males using the Chi-square test and the non-parametric bootstrapped *t*-test (function boot.*t*.test from MKinf package, 999 replication), respectively. Afterward, separate binomial generalized linear models (GLM) for each epibiont species (excluding rotifers and ciliates) were employed to investigate the effect of collection sources on epibiont prevalence while accounting for the effect of shrimp gender. These were constructed with epibiont prevalence as the dependent variable and sex and collection sources (wild, aquaculture ponds, or aquaria) as the independent variables. The results were reported as odd ratios. Similarly, habitat preferences were assessed with binomial GLMs using epibiont prevalence as the dependent variable and microhabitat (rostral area, branchial chambers, pereiopodal area, and pleopodal area) as the independent variable and the results reported as odd ratios. Possible interactions among epibionts were assessed by first selecting all shrimp with at least one epibiont recorded and then correlating epibiont abundances against each other using Spearman’s rank correlation. Spearman’s rank correlation was also employed to assess the impact of epibionts on the egg production of female shrimp and the impact of molting on epibionts.

## 3. Results

### 3.1. Identified Epibionts

Our studies led to the recognition of four morphologically different species. Two of them were consistent with the description for species belonging to two animal phyla: *Scutariella japonica* (Platyhelminthes: Temnocephalidae) and *Holtodrilus truncatus* (Annelida: Branchiobdellidae). The other two significantly differed morphologically from the species known to date. These were, therefore, separated into two new species, one relating to plants and one to animals: *Cladogonium kumaki* sp. nov. (Chlorophyta: Cladophorales: Pithophoraceae) and *Monodiscus kumaki* sp. nov. (Platyhelminthes: Rhabdocoela: Temnocephalidae). The description and redescription of recorded species is given below.

Additionally, Rotifera (Bdelloidae) and Ciliophora (Vorticellidae) were also detected.

### 3.2. Description of Species New to Science

**Kingdom Plantae**
**Phylum Chlorophyta**
**Class Ulvophyceae**
**Order Cladophorales**
**Family Pithophoraceae**

***Cladogonium kumaki***sp. nov.

**Type locality.** Asia, Taiwan, Taipei City, Wai Shuang Hsi River, 25°06′24.3″ N 121°33′27.9″ E.

**Material examined.** Individuals associated with 100 *N. davidi* shrimp.

**Holotype.** A single individual of *N. davidi* inhabited by *Cladogonium kumaki* sp. nov. is deposited in the collection of the Museum and Institute of Zoology, Polish Academy of Sciences, in Warsaw under catalog number MIZ PAN/VAR000001.

**Paratypes.** A total of five individuals of *N. davidi* with *Cladogonium kumaki* sp. nov. are deposited in the collection of the Museum and Institute of Zoology, Polish Academy of Sciences, in Warsaw under catalog numbers MIZ PAN/VAR000002 to MIZ PAN/VAR000006.

**Etymology.** The name, *Cladogonium kumaki*, is dedicated to all shrimp breeders and friends who supported the ‘Kumak Shrimp’ project, whose aim is to share knowledge relative to freshwater aquarium shrimps and, more specifically, their health issues.

**Figure 2:**
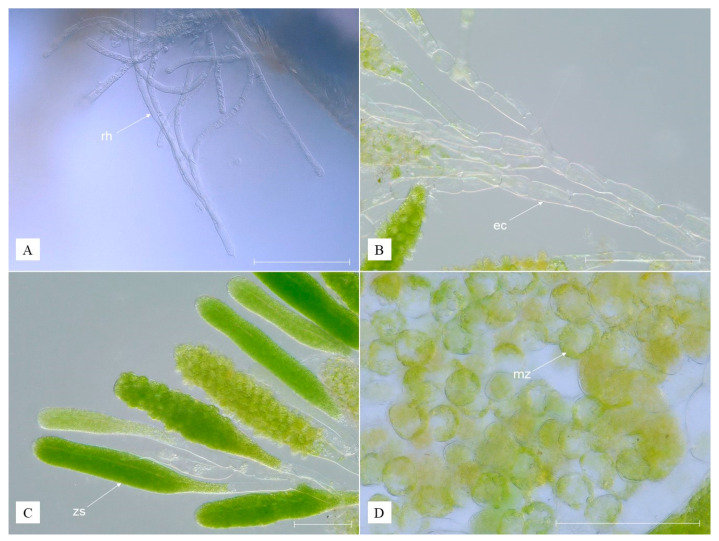
*Cladogonium kumaki* sp. nov.: portion of thallus showing several rhizoids (**A**); erect filaments (**B**); zoosporangia displaying zoospores in different stages of maturity (**C**); mature zoospores (**D**); ec—erect filament, mz—mature zoospore, rh—rhizodium, zs—zoosporangium; white scale bar = 100 µm. (Photographs by R. Maciaszek).

**Diagnosis.** Thallus is composed of an erect portion containing zoosporangia and a rhizoidal one issuing from a basal cell of an erect filament and spreading into the hypodermatic tissues of a host: rhizoidal colorless; erect cells colorless or partially light green; mature zoospores relatively big, from dark green and greenish-yellow to greenish-brown (Figure 2).

**Figure 3:**
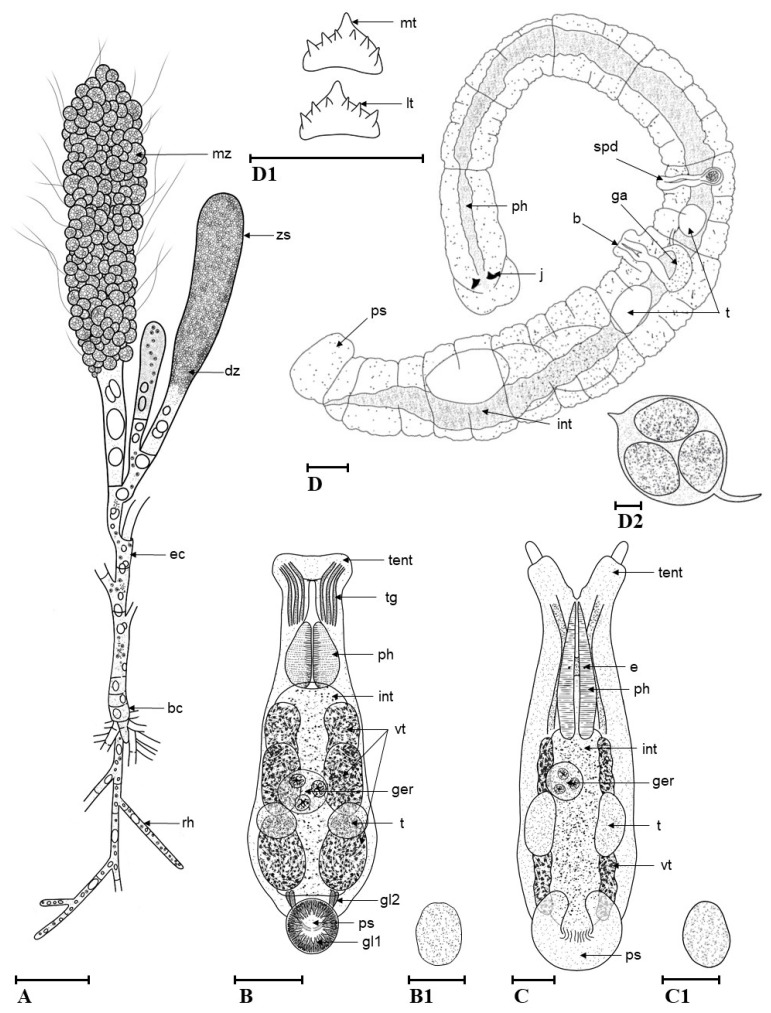
Anatomy of observed epibionts: *Cladogonium kumaki* sp. nov. (**A**), *Monodiscus kumaki* sp. nov. (**B**), *M. kumaki* sp. nov. egg (**B1**), *Scutariella japonica* (**C**), *S. japonica* egg (**C1**), *Holtodrilus truncatus* (**D**), *H. truncatus* jaws (**D1**), *H. truncatus* cocoon (**D2**); b—bursa, bc—basal cell, e—eye, ec—erect filament, dz—developing zoospore, ga—glandular atrium, ger—germarium, gl1—tiny glands of posterior sucker, gl2—large glands of posterior sucker, int—intestine, j—jaws, lt—lateral tooth, mt—median tooth, mz—mature zoospore, ps—posterior sucker, ph—pharynx, rh—rhizoidal cell, spd—spermathecal bulb, t—testis, tent—tentacle, tg—tentacular gland, vt—vitelline glands, zs—zoosporangium; black scale bar = 100 µm. (Drawings by R. Maciaszek).

**Description.** Cells of rhizodium are 6.3–9.3 µm in width and 22.1–81.9 µm in length, cylindrical, and transparent (Figure 2A). Cells of the erect filament are 7.2–36 µm in diameter and 107.5–132 µm in length, transparent, mostly colorless but sometimes partially light green depending on the quantity of chloroplasts or their traces, with four to six nuclei (Figure 2B). Zoosporangium is 19–78 µm in diameter and 322–540 µm in length, club-shaped, from almost colorless to greenish, depending on the color of zoospores in it (Figure 2C). Zoospores are ciliated. The color varies from dark green via greenish-yellow to greenish-brown in the mature zoospores, which are 19.3–35.3 µm in diameter (Figure 2D and Figure 3A).

**Ecology and distribution.** The species is a freshwater epibiotic alga living on shrimp cuticle, mostly in the pleopodal area. So far it is found only on *N. davidi* shrimp in Taiwan. However, it is likely that the species may also colonize other crustaceans, as *Cladogonium ogishimae* [25], its close relative, shows a similar ecology and was detected on different shrimp species including *Caridina leucosticte* (Stimpson, 1860), *Macrobrachium formosense* (Spence Bate, 1868), and *Paratya improvisa* (Kemp, 1917). Based on the distribution of associated hosts screened during this study, *Cladogonium kumaki* sp. nov. inhabits tropical reservoirs and watercourses, including those of anthropogenic origin, such as roadside ditches and canals. It is found in ornamental shrimp farms based on aquaculture ponds and aquariums, as well as on shrimp offered in the pet trade. It occurs in relatively warm waters (12–38 °C), slightly acidic to slightly alkaline (pH 6.0–8.2), and in a wide range of water hardness (0–27° d GH), with varying degrees of organic matter contamination. The thallus of the species provides an attachment point for other epibiotic species, including vorticellids, rotifers, temnocephalids, and *H. truncatus*.

**Remarks.***Cladogonium kumaki* sp. nov. can be distinguished from *C. ogishimae* [25], the only other known representative of the genus, by the highly irregular surface structure of mature zoosporangia and relatively big mature zoospores, which are 2.4–2.9 times bigger. Its erect filament cells are 1.2 times wider and longer; zoosporangia are 2.8 times narrower and 1.2 times shorter. A comparison, including detailed data, is presented in Table 2.

**Variability.** The quantity of the species and the size of the thallus varies between habitats. The erect portion differs in the quantity of zoosporangia containing up to 127 of them per single individual. A maximum of 695 zoosporangia were found in a single host. Among all screened hosts, the maximum number of *C. kumaki* sp. nov. found in the specific microhabitat of an individual host was, respectively, 21 in the rostral area, 93 in the branchial chambers, 12 in the pereiopodal area, and 32 in the pleopodal area. In the distinguished microhabitats, the maximum number of zoosporangia found in an individual host was, respectively, 8 in the rostral area, 42 in the branchial chambers, 222 in the pereiopodal area, and 473 in the pleopodal area. The largest number of zoosporangia per single host individual was observed in aquaculture, and the smallest was in aquaria. Although most *Cladogonium* individuals were reported to attach to shrimp in the pleopodal area, *C. kumaki* sp. nov. was also found in the other body parts of the host, including the branchial chambers and pereiopodal and rostral areas.

**Kingdom Animalia**
**Phylum Platyhelminthes**
**Class Rhabditophora**
**Order Rhabdocoela**
**Family Temnocephalidae**

***Monodiscus kumaki*** sp. nov.

**Type locality.** Asia, Taiwan, Taipei City, Wai Shuang Hsi River, 25°06′24.3″ N 121°33′27.9″ E.

**Material examined.** A total of 100 individuals associated with *N. davidi* shrimp.

**Holotype.** The holotype is deposited in the collection of the Museum and Institute of Zoology, Polish Academy of Sciences, in Warsaw under catalog number MIZ PAN/VAR000007.

**Paratypes.** A total of five individuals are deposited in the collection of the Museum and Institute of Zoology, Polish Academy of Sciences, in Warsaw under catalog numbers MIZ PAN/VAR000008 to MIZ PAN/VAR000012.

**Etymology.** The name, *Monodiscus kumaki*, is dedicated to all shrimp breeders and friends who supported the ‘Kumak Shrimp’ project, whose aim is to share knowledge relative to freshwater aquarium shrimps and, more specifically, their health issues.

**Figure 4:**
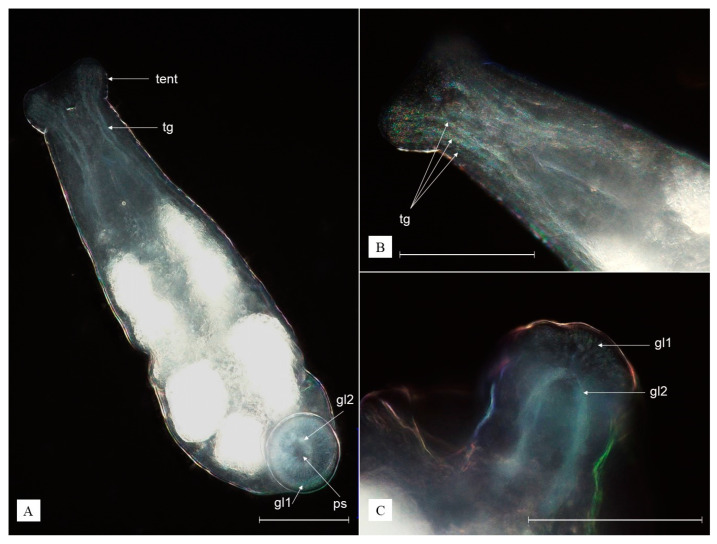
*Monodiscus kumaki* sp. nov.: ventral view (**A**), close-up image on anterior part (**B**), dorsal view on the posterior sucker (**C**); gl1—tiny glands of the sucker, gl2—large glands of the sucker; ps—posterior sucker; tent—tentacle, tg—tentacular gland; white scale bar = 100 µm. (Photographs by R. Maciaszek).

**Diagnosis.** Body is transparent white, ovoid, with two anterior tentacles and a round posterior adhesive disc (sucker); eyes absent; three sorts of tentacular glands; two types of posterior glands opening in the sucker, of which the tiny ones are concentrated at the edges of the sucker (Figure 4A).

**Description.** The body is ovoid, transparent white, reaching 0.58 mm when relaxed and over 1.21 mm when extended. Two anterior tentacles are each provided with an adhesive organ and three sorts of tentacular glands (Figure 4B). The pharynx is ovoid. Eyes are absent. The intestine lumen is not clearly visible. The vitelline glands are arranged in three groups on each side of the body, sharply pointed in bigger individuals. The genital organs are sac-like and located beneath the intestine, reaching as far as the end of the body. The penis consists of a single cuticular tube. Two ovoid testes of similar size and the ovary (germarium) are located more toward the posterior end of the body, with the latter being positioned rather in the middle and the testes on the sides. The posterior sucker is round, with openings, consisting of two types of glands: (1) tiny, evenly distributed at the edges of the adhesive organ appearing as a white halo, well visible from the ventral side; (2) large, located in the middle of the sucker (Figure 3B and Figure 4C). The eggs deposited in the host (shrimp) branchial chambers are transparent white, ovoid in shape, reaching 93–109 µm in diameter (Figure 3(B1)).

**Ecology and distribution.***Monodiscus kumaki* sp. nov. is a freshwater shrimp epibiont, living mostly in the branchial chambers of the host where it also lays eggs. It can also be found in rostral, pleopodal, and pereiopodal areas of the host, actively feeding on rotifers and protozoans. The predatory behavior of *M. kumaki* sp. nov. was commonly observed in the current study and confirmed by the presence of prey fragments visible in the digestive tract. So far, it was detected only on *N. davidi* shrimp in Taiwan and is the only known representative of *Monodiscus* living on shrimp not belonging to the genus *Caridina*. It is likely that *M. kumaki* sp. nov. may therefore colonize other atyid shrimps. Based on the distribution of infected hosts screened during this study, *M. kumaki* sp. nov. inhabits tropical reservoirs and watercourses, including those of anthropogenic origin, such as roadside ditches and canals. It is found in ornamental shrimp farms based on aquaculture ponds and aquariums, as well as on shrimp offered in the pet trade. It occurs in relatively warm waters (12–38 °C), slightly acidic to slightly alkaline (pH 6.0–8.2), a wide range of water hardness (0–27° d GH), with varying degrees of organic matter contamination.

**Remarks.***Monodiscus kumaki* sp. nov. can be distinguished from other *Monodiscus* species—first of all by the number of anterior and posterior glands. *Monodiscus kumaki* sp. nov. is characterized by three sorts of anterior, tentacular glands, while *M. parvus* has two sorts of those. On the other hand, it is distinct from *M. macbridei* by the occurrence of two instead of three types of posterior glands opening in the posterior sucker, including only big glands opening in the middle of the organ and tiny ones concentrated at its edges. Additionally, a third type of compact multicellular glands in the sucker is not present. Moreover, the mouth of *M. kumaki* sp. nov. is not lying terminally, as in *M. macbridei*, but more ventrally like in *M. parvus*. *Monodiscus kumaki* sp. nov. is also a relatively big worm compared to other species belonging to the genus. The biggest recorded individuals were almost three times bigger than representatives of *M. parvus* (0.2 mm long) and about twice as big as *M. macbridei* (0.285 mm long). The species can be distinguished from *S. japonica* by tentacles having no cylindrical base, lack of eyes, and a round posterior sucker. A detailed comparison of above-mentioned species is presented in Table 3.

**Variability.** Quantity of the species varies between habitats, reaching the highest numbers in waters full of organic matter and protozoans. A maximum of 127 individuals and 128 identified eggs were found in a single host. Among all screened hosts, the maximum number of *M. kumaki* sp. nov. found in the specific microhabitat of an individual host was, respectively, 21 in the rostral area, 93 in the branchial chambers, 12 in the pereiopodal area, and 32 in the pleopodal area. There were no significant differences in size or appearance between individuals recorded in each habitat. In smaller individuals, vitelline glands are less visible, and the body is almost completely transparent.

### 3.3. Redescription of Recorded Epibiotic Species

***Scutariella japonica*** (Matjašič, 1990)

**Synonyms.** *Cardinicola japonica* Matjašič, 1990

**Lectotype locality.** Asia, Taiwan, Taipei City, Wai Shuang Hsi River, 25°06′24.3″ N 121°33′27.9″ E.

**Material examined.** A total of 100 individuals associated with *N. davidi* shrimp.

**Lectotype (designated here).** The lectotype is deposited in the collection of the Museum and Institute of Zoology, Polish Academy of Sciences, in Warsaw under catalog number MIZ PAN/VAR000013.

**Paralectotypes.** A total of five individuals are deposited in the collection of the Museum and Institute of Zoology, Polish Academy of Sciences, in Warsaw under catalog numbers MIZ PAN/VAR000014 to MIZ PAN/VAR000018.

**Figure 5:**
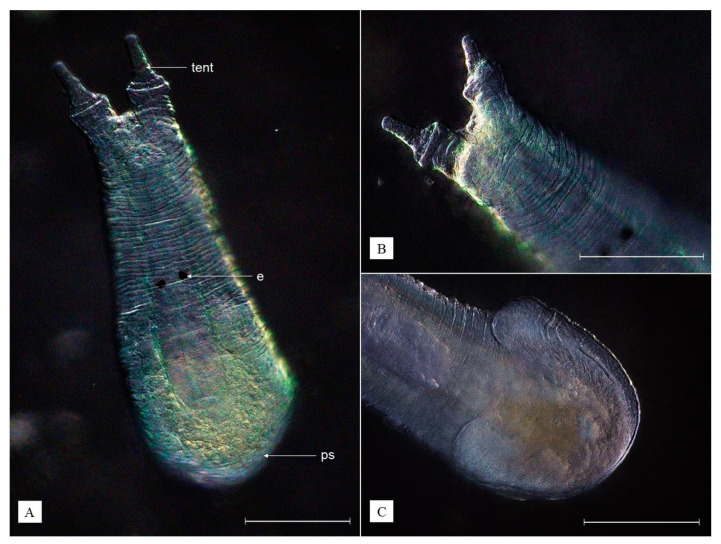
*Scutariella japonica*: dorsal view (**A**), the close-up image of the anterior part (**B**), and dorsal view on the posterior sucker (**C**); e—eye, ps—posterior sucker; tent–tentacle; white scale bar = 100 µm. (Photographs by R. Maciaszek).

**Diagnosis.** Body is transparent white, pear-shaped, flattened in its anterior part, with two tentacles and horseshoe-shaped posterior sucker; two eyes present; two testes; pharyngeal glands not well-developed (Figure 5A).

**Redescription.** Body flattened in the anterior and rounded in the posterior part, transparent white, reaching 2.65 mm when relaxed and about 3.10 mm when extended. Two tentacles are present, each with a cylindrical base and a warty terminal part (Figure 5B). Each tentacle has a sucker in its basal part and tentacular glands. The mouth opens in a small funnel-like depression between the tentacles. Pharynx is pear-shaped. Two eyes are present. Vitelline glands are located on each side of the body, sharply pointed in bigger individuals. Genital sack-like organs are located beneath the intestine, reaching as far as the end of the body. Penis consists of a single cuticular tube. Two testes and germarium are located at the posterior end of the body. The posterior sucker is horseshoe shaped (Figure 3C and Figure 5C). Eggs deposited in shrimp branchial chambers are transparent white, ovoid in shape, reaching 95–112 µm in diameter (Figure 3(C1)).

**Ecology and distribution.** *Scutariella japonica* is a freshwater atyid shrimp epibiont, living mostly in the branchial chambers of the host, where it also lays eggs. The species can often be found in the rostral, pleopodal, and pereiopodal areas of the host, feeding on rotifers and protozoans. The predatory behavior of *S. japonica* was commonly observed in the current study and confirmed by the presence of prey fragments visible in the digestive tract. To date, *S. japonica* has been found in *Neocaridina* spp. in China, Japan, and Taiwan [16,18,29,30], and more recently, Poland [17]. This epibiont has been also detected in *Caridina pseudodenticulata* (Hung et al., 1993) in Taiwan, *Caridina* sp. in China, and *Paratya compressa* (De Haan, 1844) and *P. improvisa* in Japan [18,22,29,31]. It is likely that the species may also colonize other atyid shrimps. Based on the distribution of infected hosts screened during this study, *S. japonica* inhabits tropical reservoirs and watercourses, including those of anthropogenic origin, such as roadside ditches and canals. It is found in ornamental shrimp farms based on aquaculture ponds and aquariums, as well as on shrimp offered in the pet trade. It occurs in relatively warm waters (12–38 °C), slightly acidic to slightly alkaline (pH 6.0–8.2), a wide range of water hardness (0–27° d GH), with varying degrees of organic matter contamination.

**Remarks.** Among other representatives of *Scutariella* with developed eyes, *S. japonica* is distinct from *Scutariella sinensis* (Wang and Chen, 2019) as its pharyngeal glands are not well-developed, and unlike *Scutariella indica* (Annendale, 1912), it has two instead of four testes [22]. It can be distinguished from *Monodiscus kumaki* sp. nov., as well as other *Monodiscus* species, by a horseshoe-shaped posterior sucker and the presence of eyes.

**Variability.** The quantity of the species varies between habitats, reaching the highest numbers in waters full of organic matter, as well as protozoans. A maximum of 51 individuals and 65 identified eggs were found in a single host. Among all screened hosts, the maximum number of *S. japonica* found in the specific microhabitat of an individual host was, respectively, 32 in the rostral area, 26 in the branchial chambers, 12 in the pereiopodal area, and 11 in the pleopodal area. The species presents similar sizes and appearances in each studied habitat. The vitelline glands are less visible in smaller individuals, and the body is almost completely transparent.

**Phylum Annelida**
**Class Clitellata**
**Family Branchiobdellidae**

***Holtodrilus truncatus*** (Liang, 1963)

**Synonyms.** Stephanodrilus truncatus Liang, 1963

**Lectotype locality.** Asia, Taiwan, Taipei City, Wai Shuang Hsi River, 25°06′24.3″ N 121°33′27.9″ E.

**Material examined.** A total of 100 individuals associated with *N. davidi* shrimps.

**Lectotype (designated here).** The lectotype is deposited in the collection of the Museum and Institute of Zoology, Polish Academy of Sciences, in Warsaw under catalog number MIZ PAN/VAR000019.

**Paralectotypes.** A total of five individuals are deposited in the collection of the Museum and Institute of Zoology, Polish Academy of Sciences, in Warsaw under catalog numbers MIZ PAN/VAR000020 to MIZ PAN/VAR000024.

**Figure 6:**
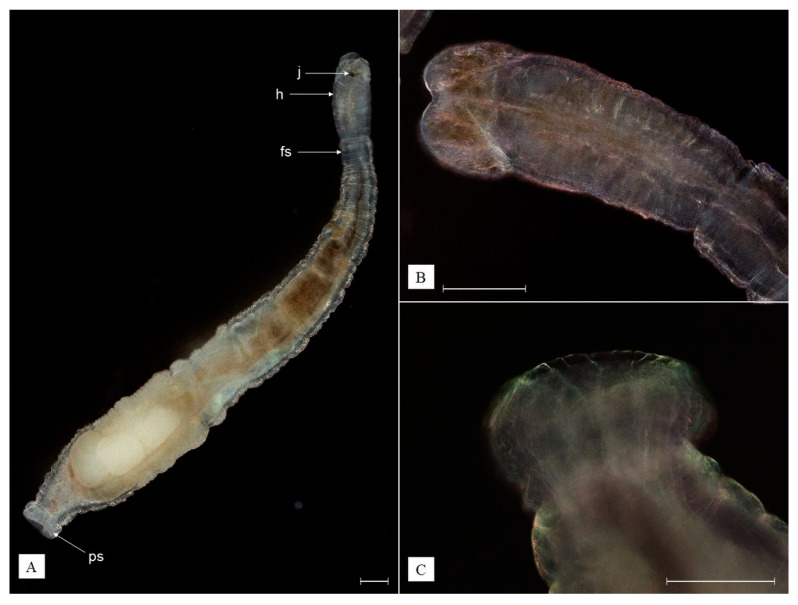
*Holtodrilus truncatus*: dorsal view of a live individual (**A**); lateral view close-up image on the head region (**B**), and lateral view posterior sucker (**C**); fs—first segment of the trunk region, j—jaws, h—head region, ps—posterior sucker; white scale bar = 100 µm. (Photographs by R. Maciaszek).

**Diagnosis.** The body is composed of 15 segments, including a distinct 4-segment head region and 11-segment trunk region, transparent, colorless or brownish, rarely spotted, with a round posterior sucker; the head region is always broader than the first segment of the trunk region and similar in size to the posterior sucker; dorsal and ventral jaws are similar in size, with 6–7 teeth each (Figure 6A,B).

**Redescription.** Body is transparent, colorless, or brownish, rarely spotted brownish, terete, reaching 2.9 mm when relaxed and 5.2 mm length when extended. Peristomal lobe and dorsal segmental appendage are absent. Mouth is surrounded by lips of which the dorsal is slightly longer than the ventral. The dorsal and ventral jaws are similar in size (25–40 µm in width) and shape: orangish-brown, triangular, with a large median tooth and three pairs of smaller lateral teeth (3-1-3/3-1-3); sometimes a single lateral tooth is missing (Figure 3(D1)). The spermathecal bulb is located in the fifth segment. A pair of testes and developing sperm are located in the fifth and sixth segments. Prostate gland is absent.

The eversible cone-shaped penis is surrounded by a muscular bursa. The posterior sucker is round, about the same diameter as the head region (Figure 3D). The cocoons comprise three to five developing embryos deposited in host branchial chambers, are transparent, ovoid in shape, reaching up to 0.6 mm in height, with a peduncle cemented onto the host gill surface (Figure 3(D2)). Fujita et al. [30] and Niwa et al. [32] reported even bigger *H. truncatus* cocoons reaching 0.76 mm and containing more developing embryos, up to 14.

**Ecology and distribution.***Holtodrilus truncatus* is a freshwater atyid shrimp epibiont, living in the branchial chambers of the host, where it also lays cocoons. The species can often be found in the pereiopodal, rostral, and pleopodal areas feeding on protozoans, rotifers, and scutariellids. The predatory behavior of *H. truncatus* was commonly observed in the current study and confirmed by the presence of prey fragments visible in the digestive tract. To date, *H. truncatus* has been found in *Neocaridina* spp. in Japan, Korea, China, and Taiwan [16,27,29,31,32], *C. pseudodenticulata* in Taiwan [26], and *C. leucostica*, *Caridina multidentata* (Stimpson, 1860), *Caridina rubella* (Fujino and Shokita, 1975), *Caridina rapaensis* (Edmondson, 1935), *Caridina typus (*H. Milne Edwards, 1937), and *P. compressa* in Japan [30,33]. It is likely that the species may also colonize other atyid shrimps. Based on the distribution of infected hosts screened during this study, *H. truncatus* inhabits tropical reservoirs and watercourses, including those of anthropogenic origin, such as roadside ditches and canals. It is found in ornamental shrimp farms based on aquaculture ponds and aquariums, as well as on shrimp offered in the pet trade. It occurs in relatively warm waters (12–38 °C), slightly acidic to slightly alkaline (pH 6.0–8.2), and in a wide range of water hardness (0–27° d GH), with varying degrees of organic matter contamination.

**Remarks.** To date, *H. truncatus* is the only known branchiobdellidan species living on Atyidae shrimp and the sole species in the genus *Holtodrilus*. It can be distinguished from *Cirrodrilus* spp. by the absence of peristomial appendages, the presence of more than two annuli per body segment, and vasa deferentia entering the ental end of the spermiducal gland [34].

**Variability.** The quantity of the species varies between habitats, reaching the highest numbers in waters full of organic matter and protozoans. A maximum of 233 individuals and 7 cocoons were found in a single host. Among all screened hosts, the maximum number of *H. truncatus* found in the specific microhabitat of an individual host was, respectively, 42 in the rostral area, 67 in the branchial chambers, 72 in the pereiopodal area, and 62 in the pleopodal area. The species presents a similar size and appearance in each habitat. The most preferred microhabitat location in shrimp is the pereiopodal area.

### 3.4. Prevalence and Mean Intensities of Epibionts

A total of 900 *N. davidi*, of which 85.4% were females and 14.6% were males, were sampled from the wild, aquaculture, and aquaria. The sex ratio among the three collection sources varied considerably (*X*^2^ (2, 900) = 9.07, *p* = 0.011). These were primarily driven by a larger proportion of females in aquaculture compared to the wild (89.7% vs. 81%, Bonferroni-corrected *p* = 0.0117). The sex ratio was more consistent between aquaria and aquaculture and between aquaria and wild (85.7% vs. 89.7%, Bonferroni-corrected *p* = 0.516 and 85.7% vs. 81% Bonferroni-corrected *p* = 0.462, respectively).

Overall, just over three-quarters of the collected shrimp were associated with at least one epibiont. Of these, females had a higher prevalence compared to males (79.3% vs. 59.5%, *X*^2^ (1, 900) = 23.24, *p* =< 0.001). This result was consistent among shrimp from the wild and aquaculture but not for those collected in aquaria (Table 4). Females also had higher epibiont mean intensity than males (Bootstrapped *t*-test, *t*(415.41) = 9.44, *p* =< 0.001). Unlike prevalence, this was consistent across all collection sources (Table 4).

The most prevalent epibionts were ciliates, followed by *H. truncatus*, *M. kumaki* sp. nov., and rotifers. When accounting for sex, the odds of hosting epibionts were highest for shrimp collected in aquaculture ponds and the lowest for shrimp obtained from aquaria. Wild shrimp had lower odds of hosting epibionts than aquaculture shrimp, except for *H. truncatus,* for which the prevalence was somewhat similar (Table 5).

### 3.5. Microhabitat Preferences, Interactions among Epibionts and Host

**Figure 7:**
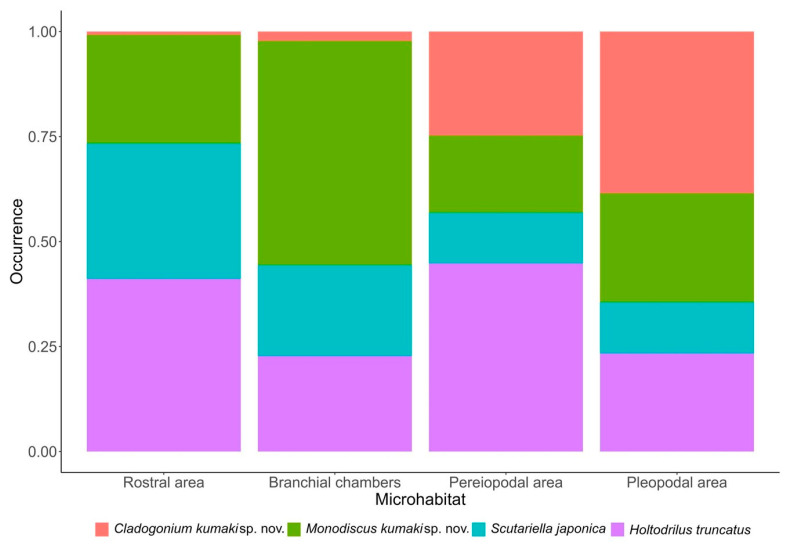
Relative proportion of epibiont species occurring in the four microhabitats.

Each epibiont species was detected in each of the four microhabitats (rostral area, branchial chambers, pereiopodal area, and pleopodal area). However, their occurrence across microhabitats differed. *Cladogonium kumaki* sp. nov. strongly preferred the pleopodal area and, in a lesser measure, the pereiopodal area. *Monodiscus kumaki* sp. nov. had marked preferences for the branchial chambers and *S. japonica* for the rostral and branchial chambers. On the other hand, *H. truncatus* preferred the pereiopodal and rostral areas in a similar measure (Figure 7, Table 6).

Despite epibionts often cooccurring in the same microhabitat, the abundance of *C. kumaki* sp. nov. appears to be from weakly to moderately positively associated with the abundance of every other epibiont. In contrast, *H. truncatus* seems to have a very small negative effect on *M. kumaki* sp. nov. and *S. japonica* (Table 7).

Among the four epibionts, only *C. kumaki* sp. nov. negatively affected egg production by female shrimp. This, however, was not strong (Spearman’s rank correlation, Rho = −0.26, *p* =< 0.001, Table 8). Molting had a low negative effect on epibiont occurrence (Spearman’s rank correlation, Rho = −0.22 to −0.31, all *p* =< 0.001, Table 8).

Molting and signs of epibiont activity after arrival in Poland: some of the shrimp went through molting. During the process, most of the examined epibionts, including all eggs and cocoons, shed together with the molt. The only exception was *C. kumaki* sp. nov. whose thallus, in some cases, partially remained attached to the host. Quickly after the molting, both temnocephalids and *H. truncatus* independently moved away from the exuvia in search of a host, often recolonizing the nearby standing original host. All of the epibionts have also been observed in some shrimp that have molted during transport.

**Figure 8:**
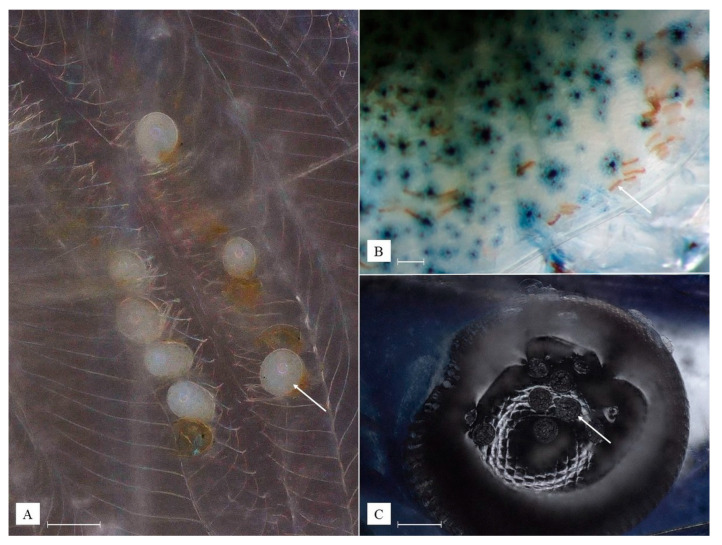
Evidence of epibiont activity: eggs of *Monodiscus kumaki* sp. nov. removed from the shrimp with its molt and visible melanization of the damaged cuticle (**A**); characteristic melanized damage in branchial chambers caused by egg laying by temnocephalids observed in live shrimp (**B**); round imprints of *Holtodrilus truncatus* on eyes and cuticle of the shrimp (**C**); white scale bar = 100 µm. (Photographs by R. Maciaszek).

Mechanical damage to the shrimp carapace was found as a result of the activity of all four selected epibionts. In the case of *C. kumaki* sp. nov., this damage resulted from thallus penetration into host tissues and its structures rubbing against other parts of the body, e.g., pleopods, pereiopods, and adjacent abdominal segments. In the rostral area, *C. kumaki* sp. nov. developed in places where the shrimp’s carapace was previously damaged. Moreover, when attached to the pleopods of shrimp incubating eggs, the thallus of *C. kumaki* sp. nov. destroyed adjacent host eggs, in some cases causing them to mold. Characteristic damages resulting from the presence of temnocephalid eggs or *H. truncatus* cocoons were found among all shrimp molts and in the branchial chambers of live individuals (Figure 8A,B). Additionally, *H. truncatus* has been observed to leave circular imprints on the carapace wherever it has been attached (Figure 8C). However, during two weeks of observations in quarantine tanks, none of this damage contributed to the deaths of the shrimp after the removal of epibionts, with the exception of cases where *C. kumaki* sp. nov. took up a significant part of the shrimp’s body. All of the damage resulting from the activity of temnocephalids and *H. truncatus* disappeared with the first molt of the shrimp.

## 4. Discussion

*Neocaridina davidi* is an ornamental shrimp commonly sold in the global aquarium trade and host of several biota. These include bacteria, ciliates, rotifers, pithophoraceans, branchiobdellidans, temnocephalids, and microsporidians [9,16,19,20,21]. If *N. davidi* is released outside its native range, cointroduction might occur with unknown impacts on native species. This phenomenon is not surprising, as *H. truncatus* or *Scutariella* sp. were already accidentally introduced into new sites together with *Neocaridina* shrimp used as live bait [16]. *Scutariella japonica* was also recently classified in the Alien Species in Poland database as potentially invasive [35] in response to its occurrence on *N. davidi* shrimp in a thermally polluted canal of the Oder River in Poland [17]. Each of examined epibiotic species may become invasive alien species posing a threat or adverse impact upon biodiversity and related ecosystem services in new sites. For example, the introduction and further spread of *S. japonica* in Europe may result in competition with *Scutariella didactyla* (Mrázek, 1907), the only known native representative of the genus living in surface waters, as well as in an impact on other epibiotic species, some of which are endemic [17]. The presence of alien epibionts poses a threat to their potential hosts, such as indigenous atyid shrimps, including *Atyaephyra desmarestii* (Millet, 1831), which may become a favorable vector because of its wide native range across the continent, coinciding with ranges of endangered species, including *Dugastella valentina* (Ferrer Galdiano, 1924) [17,24]. On the other hand, the spread of epibionts in the ornamental pet trade has been ongoing. Previous studies [9] did not indicate the presence of *H. truncatus* in aquaculture ponds in the Pingtung region of Taiwan, where it is now confirmed to be very common. Moreover, there are growing concerns among amateur aquarists about the appearance of the epibionts on shrimps [21]. This is because a particularly large accumulation of epibionts is identified by breeders as the cause of the loss of valuable coloration in ornamental shrimps, their weakening, worse breeding results, and lower crustacean survival [9].

The discovery and description of two new epibionts that seem fairly common in shrimp collected from the wild, aquaculture ponds, and aquaria, despite current veterinary inspection procedures, highlight the need for more knowledge on epibiotic fauna. Such knowledge could potentially bolster the implementation of preventive measures to curb the spread of such organisms in the international pet trade and, consequently, also lower the chances of cointroductions outside their native range. Although both new species were described here for the first time, their presence was likely detected earlier; however, they may have been misidentified. For instance, in Figure 1A of Patoka et al. [3] and Figure 4 of Maciaszek et al., [9], *Monodiscus* representatives were identified as *Caridinicola* sp. and *Scutariella japonica,* respectively. It is also possible that representatives of *Monodiscus* and *Scutariella* were both present, at least in some cases, but were recognized as representatives of a single species or genus. Such a case occurred in Liao et al., [20], where representatives of both genera were identified as *Scutariella* sp., although Figure 3B,C in their paper depict individuals of *Monodiscus*, while Figure 3D shows *Scutariella*. These can be distinguished from each other by differences in the structure of their tentacles. Such misidentifications, however, could impact previous data on occurrence and microhabitat preferences of species. A similar situation occurred in the case of *Cladogonium*, where individuals likely belonging to *C. kumaki* were identified as representatives of *C. ogishimae*, the only species known to date [9]. In addition, other authors reported cases of finding representatives of *Cladogonium* on *Neocaridina* spp. [21,36]. In each case, morphological characters of the observed individuals differed from those described in *C. ogishimae* [25], as well as in *C. kumaki*. As Matsuyama-Serisawa et al. [36] suggested, multiple species within the genus should be expected. Unfortunately, due to limited molecular sequence availability, in particular the lack of sequences of *C. ogishimae*, describing a new species within the genus *Cladogonium* is mostly possible on the basis of morphological features, which in many algae species can be plastic [37]. However, a comparison of individuals collected in different localities and in a wide range of environmental conditions can account for morphological plasticity. Such is the case in the present description, where the *Cladogonium* individuals were obtained from natural to heavily degraded ecosystems. Nevertheless, all the *Cladogonium* individuals examined in this study showed similar size and appearance irrespective of the habitat of origin, indicating low morphological plasticity. Hence, the observed morphological features of *C. kumaki* are clearly distinct from those described in *C. ogishimae*, and the requirements set out by the International Code of Nomenclature for Algae, Fungi, and Plants (Shenzhen Code) are met [38].

The taxonomic position of examined epibionts is mostly based on morphological descriptions. However, in the cases of *S. japonica* and *H. truncatus*, it is also supported by molecular data, which puts them in Platyhelminthes: Temnocephalidae and Annelida: Branchiobdellidae, respectively [18,24,39,40]. As a member of Scutariellinae, the same subfamily as *Scutariella*, the genus *Monodiscus* was placed in the family Temnocephalidae [39]. Nonetheless, the taxonomic position of *Cladogonium* remains unclear. Based on the morphological description, it was originally suggested that the genus may belong to the family Cladophoraceae within the order Cladophorales of the phylum Chlorophyta by Hirose and Akiyama [25]. Later on, it was moved to the family Pithophoraceae within the same order by Boedker et al., [41] and this taxonomic position is currently accepted [42]. However, the limited molecular data available for *Cladogonium* sp. showed that it had a closer relationship with the order Trentepohliales, with the most similar sequences not exceeding 85% similarity in the NCBI database [21]. Therefore, given the possibility of multiple species existing within the genus *Cladogonium* and its unclear taxonomic position, more comprehensive research on this genus supported with molecular data is necessary.

A higher occurrence of epibionts in shrimp collected from aquaculture ponds compared to those from wild and aquaria is not surprising. Aquaculture ponds are used for the mass production of *N. davidi* for the international pet trade. As such, shrimp are often kept in high densities and epibionts might be facilitated by the shrimp’s close proximities. Accordingly, density-dependence transmission of pathogens is a common occurrence in aquaculture [43]. Moreover, shrimp are overfed to bolster their growth and maximize profits. Hence, large quantity of organic matter may build up, boosting the availability of a wide variety of protozoan an rotifers associated with organic waste [44,45]. These might play a key role as most epibionts have been observed feeding on protozoans and rotifers [9]. Such a combination of factors is generally not present in the wild, where space availability might hinder epibiont transmission among conspecifics, nor in aquaria, where a controlled environment prevents the excessive build-up of organic matter.

A higher occurrence of epibionts in female compared to male shrimp is likely to be imputed to differences in body surface area available for attachment. Females offer a larger surface area at similar body lengths than males because of a more robust body [24]. At the same time, epibionts such as *C. kumaki* might impair egg production in female shrimp, showing its potentially parasitic behavior. Moreover, as female shrimp are larger and more colorful than males, they are typically preferred in the international pet trade [24]. Given the skewed sex ratio of traded shrimp, the risk of epibionts spreading is potentially enhanced.

*Neocaridina davidi*, like many other crustaceans, acts as a semi-permanent substrate for diverse communities of epibiotic micro-organisms. However, molting might partially or entirely remove sessile organisms as they remain attached to the cast exoskeleton [46]. Molting in *N. davidi* adversely affected the occurrence of all epibionts found in the current study. Such a process is deleterious for obligate epibionts but less so for facultative ones [47,48], which may not be easy for the crustacean to remove on its own [49]. The observations carried out as part of this work only confirm that without human intervention, the shrimp will likely not get rid of any of them on its own, even if some can be temporarily eliminated with the molt.

Various traces of epibiont activity can be recognized on the shrimp exoskeleton. They include, among others, eggs or cocoons deposited in the branchial chambers, as well as other damage resulting from the movement of epibionts or their presence. In the case of carapace damage, melanization occurs, which is perfectly visible primarily on shrimp molts. Thus, the assessment of the activity of epibionts can be carried out in a non-invasive way, solely through the observation of freshly shed molts, particularly as the molt cycle may be forced by a change in environmental conditions such as those that occurred after the import of the examined crustaceans [8,50,51]. Another noteworthy potential method of monitoring epibionts is the use of various color varieties of shrimp. Temnocephalids and branchiodbellidans occasionally feed on their hosts [22]; therefore, their internal organs might display different pigmentation depending on the host they feed upon. This can be helpful not only in observing the internal anatomy, mainly the intestine, but also in estimating the preferences of the temnocephalids for a specific host, e.g., differently colored. Moreover, different colorations might help distinguish ciliates or rotifers in the intestine of temnocephalids and *H. truncatu*s. All these activity traces can be used to determine the presence of selected epibionts and to monitor their activity and potential impact on the host and other epibiotic species.

The co-occurrence of various epibionts and their microhabitat preferences likely reflect their ecological niches and allow for cohabitation. In the present study, it was not possible to infer inter- and intraspecific competitions in terms of resource use because of limited knowledge of epibionts’ ecology and interactions. However, it is possible that certain epibionts, i.e., *C. kumaki*, might offer benefits of a mutualistic nature to other epibionts, which may lead to multifactorial disease outbreaks [21]. While others, i.e., *H. truncatus*, might antagonize the co-occurrence of further epibionts by preying on them. Both interactions can be used to control epibionts. The manual removal of *C. kumaki* might reduce the presence of other epibionts attached to its thallus. While *H. truncatus* may help control remaining epibiotic species, including temnocephalids, as shown under controlled conditions [52]. The latter may also reduce the occurrence of smaller and less visible epibionts, e.g., ciliates and rotifers, in the absence of *H. truncatus* [22]. It is also worth mentioning that previous observations showed no aggressive or defensive reactions between *H. truncatus* and *S. japonica* [31], while in this study the former was on many occasions observed hunting the latter, which may have resulted from the declining availability of other potential food. Eventually, both temnocephalids and branchiobdellidan can be eradicated using salt baths (30 s bath in 40 g sodium chloride per 1 L of habitat water) before they are finally acclimatized in the destined aquarium [8,33]. Such a solution may be particularly advantageous in control of the spread of the epibionts and might also be used for shrimp presented at competitions, where the presence of these epibiotic species is undesirable. However, with the exception of *C. kumaki*, which is likely parasitic [21], the other epibionts may regulate each other. Thus, the absence of a species might favor the establishment of others, which in the absence of competition and/or predation, can reach high numbers impairing shrimp health and even leading to host death [9,20]. Hence, their maintenance in controlled numbers, even if minor mechanical damages occur because of their activity, may actually be beneficial for shrimp, including those bred in aquaculture ponds, as is the case with ectosymbiotic branchiobdellidans in crayfish [8,49,52,53].

In a closed system, using epibionts to control other epibionts may be beneficial for shrimp and crayfish keepers. For instance, epibiotic bacteria of two commercially relevant crayfish, *Pacifastacus leniusculus* (Dana, 1852) and *Pontastacus leptodactylus* (Eschscholtz, 1823), inhibit the growth of the pathogenic oomycete *Aphanomyces astaci* Schikora, 1906, the causative agent of crayfish plague and one of the world’s 100 worst invasive alien species [54,55]. However, the use of nonpathogenic epibionts for the biocontrol of highly pathogenic ones should be exercised with caution to avoid releasing non-native epibionts into the natural environment. Moreover, shrimp and crayfish are often kept together in aquaria by both professionals and hobbyists [8]. Thus, epibionts can spill from one host to another. For instance, *H. truncatus* is known to infect other hosts including, in experimental setting, the highly invasive crayfish *P. clarkii* [32]. Unfortunately, the irresponsible release of non-native shrimp and crayfish into natural environments often entails the cointroduction of associated epibionts. Once released, epibionts might spread to native crustaceans, with unpredictable consequences. Their spread might also be facilitated by highly invasive shrimp and crayfish species, as seen for *A. astaci* [56], especially as some of these crustaceans can migrate over land [57,58,59]. Hence, preventing the release of non-native hosts and associated epibionts should be a priority not only for any responsible shrimp and crayfish keeper but also for anyone involved in the pet trade. Enhanced knowledge of epibionts and their hosts and more thorough veterinary checks of traded animals are thus essential in managing and curbing the spread of unwanted hitchhikers.

## 5. Conclusions

In conclusion, the occurrence of organisms associated with ornamental shrimps creates opportunities for their global spread via the aquarium trade. Due to small sizes, lack of coloration, and a lack of knowledge among responsible authorities, epibionts often go unnoticed. The ability to identify these species and knowledge of their microhabitat preferences and their impacts on hosts and each other can be valuable for ornamental crustacean breeders, as well as for their detection, control, and monitoring. Some epibionts may adversely affect the host, while others may be used to eliminate others. Hence, we urge more control over these epibionts to limit their spread and reduce the risk of accidental cointroductions outside their native range.

## Figures and Tables

**Table 1:** Sampling sites for wild-caught *Neocaridina davidi*.

Site Name		Coordinates	
Taipei, Taiwan	Wai Shuang Hsi River	25.0624° N	121.3327° E
	Roadside ditch 1	25.0644° N	121.3340° E
	Roadside ditch 2	25.0743° N	121.3430° E

**Table 2:** Comparison of morphological description and measurements of *Cladogonium* species.

		*Cladogonium kumaki* sp. nov. (Present Study)	*Cladogonium ogishimae* (Hirose and Akiyama, 1971)
Host species		*Neocaridina davidi*	*Caridina leucosticta* *Macrobrachium formosense* *Paratya improvisa*
Zoosporangium	Color	Shades of green	Green
	Surface structure of mature zoosporangia	Highly irregular	Rather flat
	Width in µm (mean ± SD)	19–78 (37.4 ± 19.7)	130–210 (181.5)
	Length in µm (mean ± SD)	322–540 (458.2 ± 57.5)	440–790 (561.5)
Zoospores	Color	Green	Green
	Ciliated	Yes	Yes (4)
	Diameter in µm (mean ± SD)	19.3–35.3 (26.9 ± 3.5)	8–12 μm
	Nuclei	Yes, multiple	Not determined
Erect filament of thallus	Color	Mostly colorless, sometimes partially light green	Colorless
	Width in µm (mean ± SD)	7.2–36 (26.5 ± 7.3)	17–35 (26.5)
	Length in µm (mean ± SD)	107.5–132 (120.3 ± 9.5)	40–180 (99)
	Nuclei	4–6	4–8
	Vacuolation	Yes	Yes
Rhizoidal cells	Color	Colorless	Colorless
	Width in µm (mean ± SD)	6.3–9.3 (7.2 ± 0.7)	Not determined
	Length in µm (mean ± SD)	22.1–81.9 (56.3 ± 18.6)	Not determined

**Table 3:** Comparison of morphological description and measurements of *Monodiscus* species and *Scutariella japonica*. * Morphological description and measurements based on Matjašič (1990).

	*Monodiscus kumaki* sp. nov. (This Study)	*Monodiscus parvus * (Plate, 1914) *	*Monodiscus macbridei * (Fernando, 1952) *	*Scutariella japonica * (Matjašič, 1990) *
Host species	*Neocaridina davidi* (Bouvier, 1904)	*Caridina simoni* (Bouvier, 1904)	*Caridina* sp.	*Caridina* sp. *Neocaridina* sp. *Paratya* sp.
Maximum body length when relaxed (mm)	0.58	0.20	0.285	2.65
Tentacles	Short and conical	Short and conical	Short and conical	With cylindrical base and a warty terminal part
Tentacular glands	3 sorts	2 sorts	3 sorts	
Pharynx	Ovoid	Ovoid	Ovoid	Pear-shaped
Eyes	Not present	Not present	Not present	Present
Posterior glands	2 types	2 types	3 types	
Sucker	Round	Round	Round	Horse-shoe shaped

**Table 4:** Prevalence and mean intensity of epibionts in female and male *Neocaridina davidi* from the wild, aquaculture, and aquaria. Rotifers and ciliates were excluded from mean intensity calculations.

Source	Sex	N	Epibiont Prevalence in % (*n* of Infected Individuals)						
			Pooled Epibionts	*Cladogonium* *kumaki* sp. nov.	*Monodiscus* *kumaki* sp. nov.	*Scutariella* *japonica*	*Holtodrilus* *truncatus*	Rotifers	Ciliates
Wild	F	243	86 (209)	28.8 (70)	35.4 (86)	34.6 (84)	68.3(166)	54.3(132)	73.3(178)
	M	57	54.4 (31)	1.8 (1)	7.0 (4)	8.8 (5)	47.4(27)	24.6(14)	43.9 (25)
Aquaculture	F	269	89.2 (240)	54.6 (147)	72.5 (195)	48.0 (129)	61.0(164)	61.0(164)	82.5(222)
	M	31	58.1 (18)	29.0 (9)	51.6 (16)	19.4 (6)	41.9(13)	32.3(10)	48.4 (15)
Aquaria	F	257	62.6 (161)	5.1 (13)	30.0 (77)	12.8 (33)	5.8 (15)	6.6 (17)	32.7 (84)
	M	43	67.4 (29)	4.7 (2)	25.6 (11)	18.6 (8)	11.6 (5)	7.0 (3)	34.9 (15)
Total		900	76.4 (688)	26.9 (242)	43.2 (389)	29.4 (265)	43.3 (390)	37.8 (340)	59.9 (539)
			**Epibiont mean intensity ± SE**						
Wild	F		51.6 ± 6.5	116.9 ± 13.0	6.6 ± 0.7	6.4 ± 0.8	5.2 ± 0.4	-	-
	M		5.0 ± 0.6	-	2.0 ± 0.4	3.8 ± 1.6	4.1 ± 0.5	-	-
Aquaculture	F		123.2 ± 10.7	125.0 ± 12.5	27.3 ± 1.8	11.9 ± 1.0	15.1 ± 2.3	-	-
	M		44.3 ± 11.9	40.6 ± 15.1	16.0 ± 3.9	4.7 ± 2.1	11.5 ± 3.2	-	-
Aquaria	F		44.8 ± 4.8	58.1 ± 12.6	46.7 ± 4.4	14.6 ± 2.6	3.3 ± 0.6	-	-
	M		13.2 ± 3.3	5.5 ± 0.5	19.5 ± 5.8	7.1 ± 2.1	1.6 ± 0.4	-	-
Total			73.3 ± 4.91	114.5 ± 8.7	25.6 ± 1.45	10.1 ± 0.7	9.38 ± 1.0	-	-

**Table 5:** Logistic regression output from GLMs relative to the variables shrimp source (wild and aquaria compared to aquaculture ponds) and sex (males compared to females) influencing epibiont prevalence in *Neocaridina davidi*. Confidence intervals (95%) are reported in brackets.

Variable	Odd Ratios					
	*Cladogonium kumaki* sp. nov.	*Monodiscus kumaki* sp. nov.	*Scutariella japonica*	*Holtodrilus truncatus*	Rotifers	Ciliates
Wild	0.30 (0.21–0.43)	0.19 (0.13–0.27)	0.55 (0.39–0.77)	1.33 (0.95–1.87)	0.75 (0.54–1.04)	0.60 (0.41–0.87)
Aquaria	0.05 (0.03–0.08)	0.17 (0.12–0.25)	0.20 (0.13–0.29)	0.05 (0.03–0.08)	0.05 (0.03–0.08)	0.13 (0.09–0.19)
Males	0.23 (0.12–0.42)	0.38 (0.24–0.59)	0.37 (0.21–0.60)	0.52 (0.33–0.81)	0.32 (0.20–0.52)	0.38 (0.25–0.58)

**Table 6:** Logistic regression output from GLMs’ relative microhabitat preferences (rostral, pereiopodal, and pleopodal areas compared to the branchial area) of epibionts on *Neocaridina davidi*. Confidence intervals (95%) are reported in brackets.

Variable	Odd Ratios			
	*Cladogonium* *kumaki* sp. nov.	*Monodiscus* *kumaki* sp. nov.	*Scutariella* *japonica*	*Holtodrilus* *truncatus*
Rostral area	0.28 (0.08–0.79)	0.29 (0.23–0.36)	1.27 (0.99–1.63)	1.63 (1.29–2.06)
Pereiopodal area	9.46 (5.58–17.34)	0.18 (0.14–0.23)	0.38 (0.27–0.52)	1.62 (1.28–2.05)
Pleopodal area	22.62 (13.58–40.99)	0.36 (0.29–0.45)	0.51 (0.37–0.68)	1.00 (0.78–1.29)

**Table 7:** Correlations among epibionts abundances estimated with the Spearman’s rank correlation.

Spearman’s Rank Correlation of Epibiont Abundances			
	*Cladogonium kumaki* sp. nov.	*Monodiscus kumaki* sp. nov.	*Scutariella japonica*
*Monodiscus kumaki* sp. nov.	Rho = 0.41, *p* =< 0.001		
*Scutariella japonica*	Rho = 0.39, *p* =< 0.001	Rho = 0.16, *p* =< 0.001	
*Holtodrilus truncatus*	Rho = 0.33, *p* =< 0.001	Rho = −0.16, *p* = 0.001	Rho = −0.08, *p* = 0.044

**Table 8:** Correlations between epibiont prevalence, production of eggs in female shrimp, and molting in both male and female shrimp estimated with the Spearman’s rank correlation.

Epibionts	Egg Production	Shrimp Molting
*Cladogonium kumaki* sp. nov.	Rho = −0.26, *p* =< 0.001	Rho = −0.22, *p* =< 0.001
*Monodiscus kumaki* sp. nov.	Rho = −0.05, *p* = 0.115	Rho = −0.31, *p* =< 0.001
*Scutariella japonica*	Rho = −0.07, *p* = 0.054	Rho = −0.24, *p* =< 0.001
*Holtodrilus truncatus*	Rho = −0.01, *p* = 0.847	Rho = −0.26, *p* = 0.847

## Data Availability

The data presented in this study are available on request from the corresponding author.
